# An Analysis of Cost Variation Among Drugs Available in the Indian Market for the Treatment of Chronic Bone-Related Ailments

**DOI:** 10.7759/cureus.72092

**Published:** 2024-10-22

**Authors:** Mamatha Jayachandran, Nikku M Geevarughese, Princy L Palatty, Manjeshwar Poonam Baliga-Rao, Dhanya Sacheendran, Manjeshwar S Baliga

**Affiliations:** 1 Department of Pharmacology, Amrita Institute of Medical Sciences, Amrita Vishwa Vidyapeetham, Ernakulam, IND; 2 Department of Hand Surgery, Kasturba Medical College Manipal, Manipal Academy of Higher Education, Manipal, IND; 3 Department of Pharmacovigilance, Reporting and Signal Management, Prudentia Group, New Jersey, USA; 4 Department of Research and Development, Mangalore Institute of Oncology, Mangalore, IND

**Keywords:** cost difference, cost ratio, gout, jan aushadhi, joint ailments, neck and back pain, osteoarthritis, percentage of cost variation, pharmacoeconomics, rheumatoid arthritis

## Abstract

Background: Treating widely prevalent bone illnesses such as back and neck pain, osteoarthritis, rheumatoid arthritis, gout, and osteoporosis with pharmacological medication can be excessively costly for both the individual and their family. This study aimed to conduct a cost minimization analysis by comparing the prices of Jan Aushadhi (JA) medications with the most expensive and least expensive branded alternatives available in India.

Methods: The prices of the most costly, least costly branded, and JA medicines were acquired from the relevant websites. The cost difference, cost ratio, and percentage of cost variation were determined based on the guidelines outlined in the cost minimization analysis research.

Results: A total of 67 drugs/combinations used to treat various chronic bone ailments were analyzed. With regard to analgesic/anti-inflammatory drugs, the most expensive brand of diclofenac sodium + serratiopeptidase (50mg + 10mg) tablet was 32.5 times more expensive than the cheapest and 10.5 times more costly than the JA brand. Also, the paracetamol 325mg + tramadol 37.5mg combination of the branded tablets was more expensive than JA. Branded cyclosporine was 2.3 times more expensive than JA among immunosuppressants in the disease-modifying antirheumatic drugs (DMARD) group. The most expensive immunomodulator, leflunomide, costs four times more than JA. Hydrocortisone injection, an adjuvant, costs 10.8 times more than the cheapest and 2.1 times more than JA. IL-1 inhibitor + nutraceutical combination costs 4.8 times more than the costliest JA brand. Zoledronic acid was 16.1 and 6.8 times more expensive than the cheapest branded and JA drugs. Xanthine oxidase inhibitor febuxostat, used to treat gout, costs 5.2 and 3.3 times more than JA and the cheapest brands.

Conclusion: This cost minimization study, the first to the best of our knowledge in the field of chronic musculoskeletal ailments, indicates the usefulness of JA drugs in reducing the patient's financial costs for most drugs.

## Introduction

On a global scale, a significant portion of the population is affected by chronic bone ailments arising from either inflammation or degeneration of musculoskeletal tissues [1]. The worrying aspect is that the incidence of these illnesses has globally increased from 1990 to 2017, primarily due to the rising levels of two significant risk factors, age, and obesity, and are more frequent in people above the age of 40 [1,2]. Anatomically, these ailments are classified into six subcategories: low back pain, rheumatoid arthritis, osteoarthritis, gout, neck pain, and others, which encompasses the remaining musculoskeletal disorders [3]. Physiologically, pain causes muscle stiffness and reduces mobility, leading to dependence, disability, and deformity [1]. These conditions, with various modifiable risk factors linked to their underlying pathogenesis and their sequelae, are the leading causes of disability [1]. Worse, these disorders can coexist with other age-related comorbidities (such as type 2 diabetes, hypertension, chronic obstructive pulmonary disease, chronic kidney disease, ischaemic heart disease) and can negatively affect other bodily and general health conditions, thus reducing overall quality of life considerably [1,4].

With regard to the prevalence of musculoskeletal ailments, at a global level, low back pain is the primary cause of disability, and recent reports suggest that in the year 2020, 619 million individuals were affected by it across the globe [2,4]. Projections are that this figure will rise to 843 million instances by 2050 due to population growth, the ageing of populations, and an increase in obesity [2]. The incidence of low back pain is higher in women, and its occurrence rises as individuals age, peaking at 50-55 years, and continues to increase until the age of 80 [4,5]. The association between high BMI and low back pain can be explained by body weight gain exerting localized pressure on this region's spine and tissues. In addition to this physical impact, obesity increases cytokine production, activates inflammatory processes, and aggravates low back pain [6].

Osteoarthritis, an ailment characterized by the wearing out of protective cartilage cushioning ends of the bones over time, is globally the most common bone ailment across the world. Recent reports indicate that in 2019, the global population of individuals with osteoarthritis was approximately 528 million; 73% were aged 55 years or older, and 60% were female [5]. Pathologically, osteoarthritis presents through various clinical presentations. It is usually seen to affect knees, hips, and spine, and factors like genetics, dietary habits, and obesity play an important role in the severity of the pathogenesis and its sequelae [7]. Of these, obesity is thought to be a key factor in the development and progression of hip, knee, ankle, foot, and shoulder osteoarthritic lesions [8]. Aside from the mechanical factor, inflammatory mediators produced by fat tissues harm joint cartilage, and osteoarticular overload produced by excess body weight increases the risk of osteoarthritis and bone fractures [8]. Osteoarthritis can usually be managed, although the damage to joints cannot be reversed.

Rheumatoid arthritis (RA) is a systemic autoimmune condition, and reports indicate that in the year 2020, approximately 17.6 million individuals were affected by it [2]. However, due to pharmacological advancements in newer drugs and care, the global mortality rate from RA has declined during the previous thirty years [4]. Genetic and hormone factors, stress, obesity, infections, and other factors have all been proven in the ensuing pathogenesis. Smoking is widely recognized as the primary risk factor for this morbidity, and the chemicals in cigarettes are reported to act as inflammatory mediators in the development of aggressive joint damage [9]. Although not gender-related, this effect is thought to be more pronounced in men, and age and gender differentials were not observed in the rheumatoid arthritis burden [10].

Gout is a form of arthritis marked by severe joint pain and swelling episodes. If left untreated, it can progress into chronic tophaceous gout, chronic erosive gout, or both [11]. According to recent statistics, the global prevalence of gout in 2020 was 55.8 million people, representing a 22.5% increase from 1990 [4]. The burden of gout was attributed to high BMI across different countries in both 1990 and 2017. High BMI contributes to rising gout rates among men and women. In regions with higher gout prevalence, excessive amounts of red meats, seafood, sugary foods, and beverages correlate with urate levels in the blood [11].

Osteoporosis is a condition that involves a decrease in bone mineral density and bone mass, as well as changes in bone structure and strength. These changes lead to a reduction in bone density, which increases the risk of fractures [12]. Recent reports suggest that its incidence increased by more than double from 20.3 million in 1990 to 41.5 million cases by 2019 [13]. Osteoporosis is categorized into primary (type 1 and type 2) and secondary types. Primary osteoporosis predominantly impacts post-menopausal women and individuals aged 70 and above because of the inherent process of ageing [12]. Secondary osteoporosis arises from underlying illnesses, medical interventions, or unidentified causes, and systemic disorders, endocrine diseases, and malignant neoplasms can trigger this condition. Aside from long-term use of glucocorticoids and lifestyle factors, behaviours and severe depression can also cause osteoporosis [12].

Pharmacy research relies heavily on the indispensable audit of cost minimization analysis (CMA), and one of the principal objectives in health economics and hospital pharmacy is to identify/replace medications or combinations that are both cost-effective and capable of reducing patient and societal costs [14]. Despite the importance of comprehensive and current data to determine the most effective treatments and optimal resource allocation, insufficient research attention has been given to studying the economics of treating chronic bone ailments [1,2]. Given the intricate socio-economic and healthcare obstacles in India, it is imperative to conduct a comprehensive analysis of the pharmacoeconomics of these drugs/therapies. This is essential for effectively addressing problems related to back and neck pain, osteoarthritis, rheumatoid arthritis, osteoporosis, and gout, which are significant concerns from a societal perspective [15]. The Government of India has implemented significant initiatives to ensure the affordability of essential medications for its disadvantaged populations. The purposeful initiative has enabled the general people to identify JA as an alternative among the several brands accessible across India, particularly in the most afflicted distant rural areas. In lieu of this, the objective of this study was to comprehensively examine the financial aspects of these medications, and accordingly, the financial impact of treating these illnesses has been assessed by comparing CMA, considering the costliest and cheapest branded and the Jan Aushadhi (JA) drugs.

## Materials and methods

Study design and materials

This study utilised a documented thorough pharmacoeconomic analysis methodology to evaluate the cost variations of 67 drugs/combinations used for various musculoskeletal ailments like lower back pain, osteoarthritis, rheumatoid arthritis, osteoporosis, and gout in India. The main goal was to conduct an extensive CMA, carefully comparing prices among JA, the highest-priced branded, and the lowest-priced branded medications for musculoskeletal ailments. Electronic medical records and prescriptions of patients visiting orthopedics and rheumatology outpatient departments were scrutinized to determine the list of most prescribed drugs for the earlier-mentioned musculoskeletal ailments.

Inclusion and exclusion criteria

The investigation encompassed drugs used to treat chronic musculoskeletal ailments (lower back pain, osteoarthritis, rheumatoid arthritis, osteoporosis, and gout) and appropriate supportive drugs. The drugs considered were non-steroidal anti-inflammatory drugs, biologic and non-biologic disease-modifying anti-rheumatoid drugs, anti-inflammatory drugs, antigout drugs, bone resorption inhibitors, and adjuvants used along with anti-rheumatoid medications and analgesics, like gastric-acid suppressants and nutraceuticals. Medicines used to treat other ailments were excluded from the analysis.

Data collection

The Current Index of Medical Stores (CIMS) was utilised to obtain the cost price of the drugs available in the Indian market. After a comparison of all available brands of the drugs decided to be analyzed from CIMS, the costliest and cheapest drugs were identified and taken for the study. This data was further utilised to ascertain expenses associated with the most and least costly branded pharmaceuticals across all categories in 2024. The price of JA drugs was acquired from the website of the Pharmaceuticals & Medicals Bureau of India [16].

Statistics

The cost difference (CD) was determined by subtracting the price of the less costly medication from the price of the most expensive brand. The formula utilized to compute the cost difference was

$CD = {\text{maximum cost}} - {\text{minimum cost}}$

The cost ratio (CR) was determined by dividing the maximum cost by the minimum cost. The formula employed to compute the cost ratio was

$CR = \frac{\text{maximum cost}}{\text{minimum cost}}$

In economics, fold difference describes how much a quantity changes between measurements. The concept of price variations among medications has always remained elusive to the general public. We introduced the same concept to describe the change in the costs of medicines so everyone will be able to identify the cost differences among various brands equally. Fold difference was calculated using the following formula,

\( \text{Fold difference} = \frac{\text{cost of costliest brand}}{\text{cost of cheapest brand}} \)

\( \text{Fold difference} = \frac{\text{cost of costliest brand}}{\text{cost of JA}} \)

\( \text{Fold difference} = \frac{\text{cost of cheapest brand}}{\text{cost of JA}} \)

The percentage of cost variation (PCV) is a metric that measures the degree of change in cost. The formula to create this metric represents the percentage-based cost difference between the most and least expensive branded drugs.

$PCV=\frac{\left(maximum\ cost-minimum\ cost\right)}{minimum\ cost}x\ 100$

## Results

The study analyzed the CMA (CD, CR, and PCV) for medications used to treat common musculoskeletal ailments often observed in clinics across India. The analysis encompassed a total of 89 drug formulations, and the analysis is elaborated on in Table 1. The details are enlisted as analgesic and anti-inflammatory, biologic and non-biologic DMARDs, anti-inflammatory (IL-1 inhibitor), antigout drugs (xanthine oxidase inhibitor, uricosuric agent, anti-inflammatory), bone resorption inhibitor (bisphosphonates), adjuvants used along with anti-rheumatoid medications and analgesics, like gastric-acid suppressants (H2 receptor blocker, proton pump inhibitors) and nutraceuticals (vitamin D) (Table 1).

**Table 1:** Comparison of cost minimization analysis of branded costliest and cheapest drugs, with Jan Aushadi drugs for musculoskeletal ailments in India CD20: B-lymphocyte antigen CD20, H2: histamine receptor H2, IL-1: interleukin-1, INR: Indian rupees, IV: intravenous, JA: Jan Aushadhi, mcg: microgram, mg: milligram, ml: milliliter, NA: not available, TNF: tumor necrosis factor, vs: versus, w/w: weight for weight, WHO: world health organisation.

Therapeutic category		Generic name of the medicine	Drug dose	Cost per unit (in INR)			Cost difference (in INR)			Fold difference			Percentage of cost variation		
				JA	Costly	Cheap	Costly vs cheap	Costly vs JA	Cheap v. JA	Costly vs cheap	Costly vs JA	Cheap vs JA	Costly vs cheap	Costly vs JA	Cheap vs JA
Analgesic and anti-inflammatory	Step 1 WHO pain ladder	Aceclofenac + paracetamol tablet	100mg + 325mg	1	9.00	2.8	6.2	8	1.8	3.2	9	2.8	221.4	800	180
		Aceclofenac 100mg + paracetamol 325mg + serratiopeptidase 15mg tablet	100mg + 325mg + 15mg	1.9	12.50	6.4	6.1	10.6	4.5	2	6.6	3.4	95.3	557.9	236.8
		Aceclofenac 100mg + paracetamol 325mg + thiocolchicoside 4mg tablet	100mg + 325mg + 4mg	5	19.40	14.195	5.2	14.4	9.2	1.4	3.9	2.8	36.7	288	183.9
		Aceclofenac tablet	100mg	0.8	4.82	1.45	3.4	4	0.6	3.3	6	1.8	232.9	502.5	81
		Aceclofenac sustained release 200mg tablet	200mg	2	7.57	3	4.6	5.6	1	2.5	3.8	1.5	152.5	278.8	50
		Acetylsalicylic acid (aspirin) enteric-coated tablet	325mg	0.57	0.95	0.25	0.7	0.4	-0.3	3.8	1.7	0.4	277	66.7	-55.8
		Celecoxib tablet	100mg	0	12.11	2.1	10	12.1	2.1	5.8	NA	NA	476.7	NA	NA
		Celecoxib tablet	200mg	0	20.95	4.17	16.8	21	4.2	5	NA	NA	402.4	NA	NA
		Diclofenac gel (diclofenac diethylamine 1.16 % w/w)	1.16% w/w 15g	12	50.25	28.5	21.8	38.3	16.5	1.8	4.2	2.4	76.3	318.8	137.5
		Diclofenac potassium 50mg + paracetamol 325mg + serratiopeptidase 10mg tablet	50mg + 325mg + 10mg	1.98	13.80	4.80	9	11.8	2.8	2.9	7	2.4	187.4	596.8	142.4
		Diclofenac sodium gastro-resistant Tablet	50mg	0.55	6.27	2.08	4.2	5.7	1.5	3	11.4	3.8	201.3	1039.3	278.2
		Diclofenac Sodium prolonged-release tablet	100mg	1.21	9.86	2.50	7.4	8.7	1.3	3.9	8.1	2.1	294.4	714.9	106.6
		Diclofenac sodium + serratiopeptidase tablet	50mg + 10mg	1.54	16.23	0.50	15.7	14.7	-1	32.5	10.5	0.3	3146	953.9	-67.5
		Etoricoxib tablet	120mg	3.8	15.9	7.25	8.7	12.1	3.5	2.2	4.2	1.9	119.3	318.4	90.8
		Etoricoxib tablet	90mg	3.1	16.5	4.95	11.6	13.4	1.9	3.3	5.3	1.6	233.3	432.3	59.7
		Ibuprofen tablet	200mg	0.3	0.67	0.36	0.3	0.4	0.1	1.9	2.2	1.2	86.1	123.3	20
		Ibuprofen tablet	400mg	0.8	0.91	0.67	0.2	0.1	-0.1	1.4	1.1	0.8	35.5	14.1	-15.8
		Ketoprofen tablets	50mg	0	22.1	0.7	21.4	22.1	0.7	31.6	NA	NA	3057.1	NA	NA
		Naproxen tablet	250mg	1.53	8.89	2.97	5.9	7.4	1.4	3	5.8	1.9	199.3	481	94.1
		Naproxen tablet	500mg	3.2	13.6	6.11	7.5	10.4	2.9	2.2	4.3	1.9	122.6	325	90.9
		Paracetamol 325mg + diclofenac sodium 50mg tablets	325mg + 50mg	0.99	6.13	0.95	5.2	5.1	0	6.4	6.2	1	544.8	518.8	-4
		Paracetamol tablet	650mg	1	2.28	1.66	0.6	1.3	0.7	1.4	2.3	1.7	37.3	128	66
		Paracetamol tablet	1000mg	1.6	4.57	2.87	1.7	3	1.3	1.6	2.8	1.8	58.8	184.9	79.4
		Piroxicam injection 20mg in 1ml	20mg	6	36	4.2	31.8	30	-1.8	8.6	6	0.7	757.1	500	-30
		Piroxicam dispersible tablets 20 mg	20mg	2.1	8.8	3	5.8	6.7	0.9	2.9	4.2	1.4	193.3	319	42.9
	Step 2	Paracetamol 325mg + tramadol 37.5mg tablets	325mg + 37.5 mg	1.32	12	4.2	7.8	10.7	2.9	2.9	9.1	3.2	185.7	809.1	218.2
Disease-modifying anti-rheumatoid drugs: non-biologics (immunosuppressant)		Cyclosporine capsules	50mg	24	57.40	56.8	0.6	33.4	32.8	1	2.4	2.4	1.1	139.2	136.7
		Cyclosporine capsules	100mg	46	106.6	106.2	0.4	60.6	60.2	1	2.3	2.3	0.4	131.7	130.9
		Methotrexate tablets	2.5mg	1.5	5.27	1.89	3.4	3.8	0.4	2.8	3.5	1.3	179.5	251.4	25.7
		Methotrexate tablets	7.5mg	2.5	12.19	11.42	0.8	9.7	8.9	1.1	4.9	4.6	6.7	387.6	357
Disease-modifying anti-rheumatoid drugs: non-biologics (immunomodulators)		Hydroxychloroquine	400mg	8.2	15.1	11.50	3.6	6.9	3.3	1.3	1.8	1.4	31.3	84.1	40.2
		Hydroxychloroquine	200mg	5.59	5.91	5.89	0	0.3	0.3	1	1.1	1.1	0.3	5.6	5.3
		Leflunomide tablets	20mg	7.4	29.3	13.80	15.5	21.9	6.4	2.1	4	1.9	112.3	295.9	86.5
Disease-modifying anti-rheumatoid drugs: biologics (TNF-alpha inhibitor)		Adalimumab injection	40mg 0.8 ml prefilled syringe	13000	26353	18000	8353	13353	5000	1.5	2	1.4	46.4	102.7	38.5
		Etanercept injection	50mg	0	16646	7000	9646	16646	7000	2.4	NA	NA	137.8	NA	NA
		Infliximab injection	100mg	0	32000	20519	11481	32000	20519	1.6	NA	NA	56	NA	NA
Disease-modifying anti-rheumatoid drugs: biologics (CD20 inhibitor)		Rituximab 100mg/10ml injection	100mg/10ml	0	8530	6108.1	2421.9	8530	6108.1	1.4	NA	NA	39.7	NA	NA
		Rituximab 500mg/50ml injection	500mg/50ml	0	42650	34123.3	8526.7	42650	34123.3	1.2	NA	NA	25	NA	NA
Antirheumatoid drugs: Adjuvants		Dexamethasone 2ml injection	4mg IV	6	11.5	7.12	4.4	5.5	1.1	1.6	1.9	1.2	61.5	91.7	18.7
		Hydrocortisone sodium succinate injection	100mg IV	20	41.41	3.84	37.6	21.4	-16.2	10.8	2.1	0.2	979.8	107.1	-80.8
		Dexamethasone tablet	0.5mg oral	0	0.39	0.20	0.2	0.4	0.2	1.9	NA	NA	93.1	NA	NA
Anti-inflammatory: IL-1 inhibitor		Diacerein capsules	50mg	4.5	15.57	5.59	10	11.1	1.1	2.8	3.5	1.2	178.8	246.1	24.2
Anti-inflammatory: IL-1 inhibitor + nutraceutical		Diacerein + glucosamine sulfate tablet	50mg + 500mg	5.5	16.0	14.48	1.5	10.5	9	1.1	2.9	2.6	10.5	190.9	163.3
		Diacerein 50 mg + methyl-sulphonyl-methane 250 mg + glucosamine sulphate 750mg tablet	50mg + 250mg + 750mg	4.95	24.0	10.6	13.4	19.1	5.7	2.3	4.8	2.1	126.4	384.8	114.1
Bone resorption inhibitor: bisphosphonates		Zoledronic acid injection	4 mg per 5ml	200	3212	1357.5	1854.5	3012	1157.5	2.4	16.1	6.8	136.6	1506	578.7
		Alendronate 70mg tablet	70mg	5	78.165	22.5	55.7	73.2	17.5	3.5	15.633	4.5	247.4	1463.3	350
		Risedronate 35mg tablet	35mg	NA	97.5	23.55	73.9	NA	NA	4.1	NA	NA	314.0	NA	NA
		Ibandronic acid 150mg tablet	150mg	325	430	15	415.0	105.0	-310	28.7	1.3	0.04	2766.7	32.3	-95.4
Parathyroid hormone compounds		Teriparatide 750mcg injection	750mcg	3269	9999	5989.5	4009.5	6730	2720.5	1.7	3.1	1.8	66.9	205.9	83.2
Antigout drug: xanthine oxidase inhibitor		Allopurinol tablet	100mg	1.1	3.60	1.86	1.7	2.5	0.8	1.9	3.3	1.7	93.9	227.3	68.8
		Allopurinol tablet	300mg	3	8.19	7.11	1.1	5.2	4.1	1.2	2.7	2.4	15.3	173.1	136.8
		Febuxostat tablets	40mg	2	13.5	5.40	8.1	11.5	3.4	2.5	6.8	2.7	150	575	170
		Febuxostat tablets	80mg	3.4	17.6	11.20	6.4	14.2	7.8	1.6	5.2	3.3	57.1	417.6	229.4
Antigout: uricosuric agent		Probenecid tablet	500mg	NA	6.8	0	6.8	6.8	0	NA	NA	NA	NA	NA	NA
Antigout: anti inflammatory		Colchicine tablet	0.5mg	NA	3.47	3.27	0.2	3.5	3.3	1.1	NA	NA	6	NA	NA
Gastric acid suppressants: H2 receptor blocker		Cimetidine tablet	200mg	NA	2.76	0.72	2	2.8	0.7	3.8	NA	NA	283.3	NA	NA
		Ranitidine tablets	150mg	0.6	0.52	0.48	0	-0.1	-0.1	1.1	0.9	0.8	8.7	-12.7	-19.7
		Ranitidine tablets	300mg	1.3	1.96	1.76	0.2	0.7	0.5	1.1	1.5	1.4	11.4	51	35.6
Gastric acid suppressants: proton pump inhibitors		Esomeprazole tablets	40mg	2.09	10.7	5.50	5.2	8.6	3.4	1.9	5.1	2.6	94.5	412	163.2
		Lansoprazole capsules	15mg	2	7.32	2.86	4.5	5.3	0.9	2.6	3.7	1.4	156.2	266	42.9
		Omeprazole gastro-resistant capsules	20mg	0.99	4.29	1	3.3	3.3	0	4.3	4.3	1	328.6	332.9	1
		Omeprazole 20mg and domperidone 10mg capsules	20mg + 10mg	1.1	6.69	3.84	2.9	5.6	2.7	1.7	6.1	3.5	74.3	508.3	248.9
		Pantoprazole gastro-resistant tablets	40mg	1.21	16.5	6.24	10.3	15.3	5	2.6	13.6	5.2	164.4	1263.6	415.7
		Pantoprazole 20mg and domperidone 10mg capsules	20mg + 10mg	2	11	4.1	6.9	9	2.1	2.7	5.5	2.1	168.3	450	105
		Pantoprazole 40mg (gastro-resistant) and domperidone 30mg (prolonged release) capsules	40mg + 30mg	2.2	10.23	5.4	4.8	8	3.2	1.9	4.7	2.5	89.5	365	145.5
		Rabeprazole gastro-resistant tablets 20mg	20mg	0.88	16.6	2.5	14.1	15.7	1.6	6.6	18.9	2.8	564	1786.4	184.1
Nutraceuticals: vitamin D		Alfacalcidol soft gelatin capsules	0.25 mcg	2.2	13.58	5.54	8	11.4	3.3	2.5	6.2	2.5	145.1	517.3	151.8

67 drugs/formulations were analyzed in the analgesic/anti-inflammatory category. Celecoxib tablets (100 and 200mg) and ketoprofen (50mg) were unavailable in JA stores. The most significant cost differences were observed between the most expensive brand of diclofenac sodium + serratiopeptidase tablet (50mg + 10mg), which was 32.5 times more expensive than the cheapest and 10.5 times more costly than the JA brand (INR 16.23 vs INR 1.54 vs INR 0.50) (Table 1). In addition to this, results also suggest that the cost difference was significant per tablet between the costliest (INR 12.00) vs cheapest (INR 4.20) vs JA (INR 1.32) for the combination of paracetamol 325mg + tramadol 37.5mg tablets (Table 1). Among the individual drugs, a big difference in cost was observed for piroxicam dispersible tablets of 20 mg strength with prices of INR 8.80, INR 3.00, and INR 2.10 observed per tablet of the costliest, cheapest, and JA brands, respectively (Table 1).

In the DMARD category, cyclosporine was 2.3 times costlier compared to the costliest and cheapest brands of JA in immunosuppressants. In contrast, the costliest leflunomide was four times costlier than JA in immunomodulators. Identifying the only JA drug among TNF-alpha inhibitors, adalimumab injection was found to be two times cheaper compared to the costliest brand of the same medication. No JA alternatives were available for the CD20 inhibitor DMARD rituximab. The bisphosphonate zoledronic acid was 16.1 and 6.8 times costlier, comparing costliest to JA and cheapest to JA brands, respectively.

In gout treatment, the xanthine oxidase inhibitor febuxostat was found to be 5.2 times more expensive when comparing the costliest brand to the JA brand and 3.3 times more expensive when comparing the cheapest brand to the JA brand. At the same time, colchicine and uricosuric probenecid did not have a JA counterpart. The generic alternatives for certain medications like colchicine and probenecid are unavailable in JA stores, so patients will have to choose their branded economic counterparts that are available in regular drug stores. We have not focused on non-JA generic drugs as our focus was the comparison between the costliest brands vs cheapest brands vs JA.

Regarding the adjunct drugs used to mitigate adverse effects of the standard medications used for musculoskeletal ailments, the analysis for gastric protective drugs indicated that ranitidine 300mg, the costliest brand, was 1.5 times costlier than the JA brand. In contrast, pantoprazole and rabeprazole demonstrated a change of 13.6 and 18.9 folds, respectively. Hydrocortisone sodium succinate injection used as an adjuvant was 10.8 times more expensive than the cheapest and 2.1 times more costly than JA. The costliest brand of IL-1 inhibitor + nutraceutical combination was 4.8 times costlier than JA (Table 1). The cost of Vitamin D supplements showed a 6.2-fold increase when comparing the costliest brand to the JA brand and a 2.5-fold increase when comparing the cheapest brand to the JA brand (Table 1).

## Discussion

Chronic ailments necessitate the regular use of medication, doctor’s visit, hospitalisation, testing, and long-term care, all of which causes significant expenses [17]. A large share of direct spending goes to medications, mostly pain-related and DMARDs. At the same time, work-related productivity losses and home care costs, in the worst cases, account for the indirect costs [1]. In this situation, patients may experience exorbitant out-of-pocket expenses that hinder prompt and adequate medical care, abstinence from drug intake, and doctor’s follow-up care visits, aggravating musculoskeletal and general health conditions [18]. In India, where much of the population lives in poverty, it is crucial to strike a careful equilibrium between effective healthcare, ensuring economic viability, and supporting self and family [19].

With regard to the drugs analyzed, analgesic drugs with anti-inflammatory effects are the most prescribed medications for a wide range of bone ailments, and their wide usage has contributed to formulating a wide range of formulation combinations for enhanced therapeutic efficacy. The economic challenges of analgesic drugs arise from their demand and the multiple companies that produce and market them (Table 1). Currently, the WHO pain ladder is utilized to manage a variety of chronic pain conditions associated with degenerative, musculoskeletal, and cancer [20]. The narrower cost differences observed in DMARDs seem to be influenced by factors such as targeted applications, specific disease profiles, and perhaps a more standardized market for these categories. Anti-gout and anti-rheumatoid drugs have particular pharmacological uses and are not prescribed to treat a wide range of bone ailments, resulting in a more uniform cost distribution within these categories (Table 1).

The results of the study affirmed that generic drugs are generally cheaper than branded ones. As far as the authors are aware, this is the first study to the best of our knowledge that addresses the cost analysis and price variability of branded medicines of JA drugs marked for chronic bone ailments. However, previous studies have shown that JA drugs are cost-effective for psychiatric medications [21], psychotropic medicines [22], anticancer drugs for head and neck cancer [23,24], breast cancer [25], lung cancers [26], type 2 diabetes [27], and thromboembolic disorders [28]. This has major implications for improving access to important pharmaceuticals, especially for economically deprived groups. JA medicines' economic benefits support the goal of making healthcare more accessible and reducing patient costs.

This study addresses the cost analysis and price variability of branded medicines of JA drugs for chronic bone ailments. The findings from this study revealed substantial cost variations in the drugs used for treating bone ailments. The biggest drawback of the study is that the attempt has been only focused on cost aspects and not on the relative efficacy and the degree of financial burden to the patient, especially of a marginal background. Studies are planned to be undertaken in the future, covering these aspects as they will give a clear idea of the total efficacy and usefulness to patients and society.

Limitations

The biggest drawback of the study is that the attempt has been only focused on cost aspects and not on the relative efficacy and the degree of financial burden to the patient, especially of a marginal background. Studies are planned to undertake these aspects as they will give a clear idea of the total efficacy and usefulness to patients and society.

## Conclusions

The findings of the study revealed substantial pricing variations among the different kinds of drugs used for bone ailments. The economic impact of bone illness medication has wide-ranging consequences on healthcare systems. This observation offers substantial evidence regarding the costs associated with medications in India. The study's conclusions are significant for subsequent deliberation on the topic by the general public, legislators, and healthcare providers. From a social standpoint, the use of generic drugs can significantly reduce the economic burden connected with healthcare. Considering this, healthcare professionals should promote the utilisation of generic pharmaceuticals, especially JA drugs, by prescribing and dispensing them while also clarifying any misunderstandings that patients may hold. Policymakers and healthcare professionals can utilize these data to advocate for the adoption of generic medicine to establish a healthcare system that is both sustainable and cost-effective.
